# Effects of Storage Temperatures on Nitrogen Assimilation and Remobilization during Post-Harvest Senescence of Pak Choi

**DOI:** 10.3390/biom13101540

**Published:** 2023-10-18

**Authors:** Savitha Dhandapani, Vidya Susan Philip, Shaik Anwar Ahamed Nabeela Nasreen, Alice Mei Xien Tan, Praveen Kumar Jayapal, Rajeev J. Ram, Bong Soo Park

**Affiliations:** 1 Temasek Life Sciences Laboratory, National University of Singapore, 1 Research Link, Singapore 117604, Singapore; savitha@tll.org.sg (S.D.);; 2 Disruptive & Sustainable Technologies for Agricultural Precision, Singapore-MIT Alliance for Research and Technology, 1 CREATE Way, Singapore 138602, Singapore; 3 Research Laboratory of Electronics, Massachusetts Institute of Technology, Cambridge, MA 02139, USA

**Keywords:** post-harvest, shelf life, dark-induced leaf senescence, nitrogen remobilization, storage temperature, Pak Choi, leafy vegetables

## Abstract

In the agricultural industry, the post-harvest leafy vegetable quality and shelf life significantly influence market value and consumer acceptability. This study examined the effects of different storage temperatures on leaf senescence, nitrogen assimilation, and remobilization in Pak Choi (*Brassica rapa* subsp. *chinensis*). Mature Pak Choi plants were harvested and stored at two different temperatures, 4 °C and 25 °C. Senescence was tracked via chlorophyll content and leaf yellowing. Concurrently, alterations in the total nitrogen, nitrate, and protein content were quantified on days 0, 3, 6, and 9 in old, mid, and young leaves of Pak Choi plants. As expected, 4 °C alleviated chlorophyll degradation and delayed senescence of Pak Choi compared to 25 °C. Total nitrogen and protein contents were inversely correlated, while the nitrate content remained nearly constant across leaf groups at 25 °C. Additionally, the transcript levels of genes involved in nitrogen assimilation and remobilization revealed key candidate genes that were differentially expressed between 4 °C and 25 °C, which might be targeted to extend the shelf life of the leafy vegetables. Thus, this study provides pivotal insights into the molecular and physiological responses of Pak Choi to post-harvest storage conditions.

## 1. Introduction

Nitrogen (N) functions as a primary nutrient and serves a critical role in the life cycle of plants. It is essential for standard plant growth, development, and stress responses [1]. N is predominantly absorbed by roots in the form of nitrate and ammonium ions [2]. During the vegetative stage, plant leaves act as a N sink and temporarily store N in their vacuoles, whereas, during leaf senescence, stored N is remobilized and reutilized for the growth of younger tissues [3,4]. N is one of the most important elements mobilized during leaf senescence, with its concentration reduced by up to 80% in senescent leaves [5]. 

Leaf senescence is the final phase of leaf development and is characterized by the loss of chlorophyll, leaf yellowing, and degradation of chloroplast proteins [6]. Since chloroplasts contain 70% of leaf N, amino acids and nucleic acids released by their degradation are also the main sources of N remobilization [7,8]. Several nitrate transporters, ammonium transporters, and amino acid transporters are upregulated during leaf senescence [3]. While the *aap2*, *aap3*, and *aap8* mutants of amino acid transporter genes exhibited delayed leaf senescence [9,10,11], the overexpression of *NRT1.1*/*NPF6.3* led to delayed leaf yellowing under N-deficient conditions [12]. While there is abundant research covering the role of N uptake, assimilation, and remobilization in natural leaf senescence, knowledge about the same during post-harvest leaf senescence remains vastly undiscovered. Uncovering the role of N assimilation and remobilization during post-harvest leaf senescence might prove useful for improving the post-harvest quality and shelf life of leafy vegetables.

Leafy vegetables are highly perishable products and undergo significant nutritional loss during post-harvest storage. A comprehensive examination of the nutrient composition and visual quality of lettuce during post-harvest storage revealed a remarkable decrease in bioactive and antioxidant compounds, accompanied by deteriorated visual appeal [13]. Thus far, a few approaches have been reported to boost the quality and shelf life of post-harvest leafy vegetables. Low O_2_ with high CO_2_ treatment enhanced carotenoid content without compromising the visual quality of lettuce, spinach, and corn salad [14]. In another study, supplemental blue light boosted the production of secondary metabolites, while red light effectively preserved phytochemicals in baby-leaf lettuce [15]. Exogenous melatonin treatment delayed postharvest senescence of broccoli, cabbage, and baby mustard [16,17,18]. Interestingly, increasing the concentration of abscisic acid or its partial agonist pyrabactin from the beginning of the post-harvest period could also effectively slow down the senescence of the stored leaves [19].

Pak Choi (*Brassica rapa* subsp. *chinensis*) is one of the most important leafy vegetables in China and represents 30–40% of the total vegetable consumption [20]. This fast-growing leafy vegetable is rich in various minerals and vitamins, including fiber, iron, calcium, and vitamin A. Beyond its high nutritional content, Pak Choi is esteemed for its mild flavor and versatility. Its economic significance is only growing due to its surging popularity in Europe and the Midwest in recent years. However, post-harvest loss poses a significant challenge for Pak Choi farmers. Similar to other leafy vegetables, prolonging the shelf life and preserving the quality of harvested Pak Choi is challenging, as its leaves lose water and turn yellow during post-harvest storage, making it unmarketable. Techniques that can mitigate post-harvest leaf senescence will improve the shelf life of Pak Choi, reducing economic losses.

Hence, the primary objective of this study was to understand the status of N assimilation and remobilization during the post-harvest leaf senescence in Pak Choi. By investigating this crucial aspect of plant nutrient dynamics, we aim to fill the current knowledge gap and its potential implications for plant biology and the food industry.

## 2. Materials and Methods

### 2.1. Plant Material and Growth Conditions

Seeds of Pak Choi (*Brassica rapa* subsp. *chinensis*) were sterilized and sown on an agar medium containing Murashige and Skoog (MS) basal salts, 1% (*w*/*v*) sucrose, and 2.5 mM MES (Sigma-Aldrich, St. Louis, MO, USA), pH 5.6. After undergoing imbibition at 4 °C in the dark for 2 days, seedlings were germinated in a tissue culture chamber at 22 °C with 60% relative humidity under 16 h light/8 h dark condition (100 µmol·m^−2^·s^−1^ light intensity). After 7 days, 42 seedlings were transplanted to seven hydroponic trays, each containing 3 L of Hoagland’s solution, and were placed in a growth chamber at 22 °C with 60% relative humidity under 16 h light/8 h dark condition (100 µmol·m^−2^·s^−1^ light intensity). The nutrient solution was renewed every 4 days.

### 2.2. Analysis of Post-Harvest Leaf Senescence

After 4 weeks, six plants were first harvested (day 0, D0). Twelve leaves from each plant were grouped as old (leaves 1–4; from bottom), mid (leaves 5–8), and young (leaves 9–12) and used for all assays. The four leaves in each group were arranged on top of each other, and leaf discs were punched out and frozen in liquid nitrogen for qPCR analysis. Similarly, leaf discs were taken from multiple locations for total chlorophyll, nitrate, and total protein assays from old, mid, and young leaves. Leaf discs made from the four leaves were pooled as a single replicate. The remains of the leaves from each group were dried in individual glass beakers at 65 °C for total N analysis.

**Figure 1:**
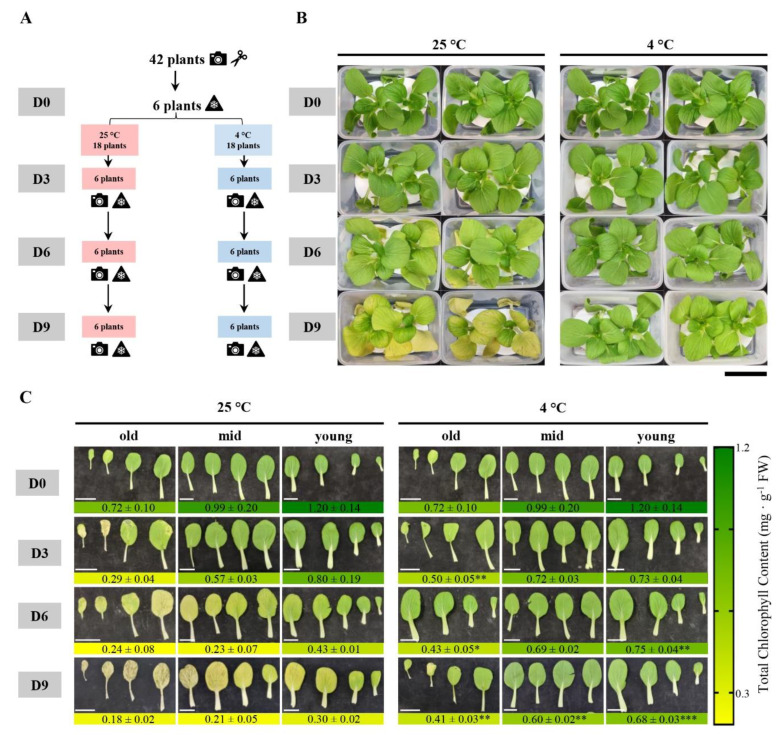
Effect of the post-harvest storage temperature on leaf senescence in Pak Choi. (**A**) A flowchart describing the post-harvest dark treatment conditions and durations. The images 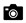, 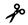, and 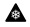 indicate imaging, harvesting, and freezing of plant/leaf samples, respectively. (**B**) Morphological phenotype of the Pak Choi plants on the harvesting day (D0) and on day 3 (D3), day 6 (D6), and day 9 (D9) after storage at 4 °C and 25 °C under dark conditions. Plants stored at 4 °C showed a delayed dark-induced senescence and remained green. Scale bar, 10 cm. (**C**) Images (top) and total chlorophyll content (bottom) of the leaves categorized as old (leaves 1–4), mid (leaves 5–8), and young (leaves 7–12) on D0, D3, D6, and D9. Scale bars, 5 cm. The treatments were carried out thrice, and the representative images from these three independent experiments are shown. For total chlorophyll analysis, data are mean ± SD, n = 6. The statistical significance of the 4 °C treatment compared to the 25 °C treatment was determined by a two-tailed *t*-test (* *p* < 0.05, ** *p* < 0.01, and *** *p* < 0.001). The *p*-values are given in Supplementary Table S2.

Next, the remaining 36 plants were harvested, divided into two groups, and then stored under darkness at 4 °C and 25 °C, as shown in Figure 1A. On day 3 (D3), day 6 (D6), and day 9 (D9), six plants were retrieved from 4 °C and 25 °C and imaged, and the leaves were processed as described above for various analysis.

### 2.3. Determination of Total Chlorophyll Content

The total chlorophyll content in the leaves was determined as described previously [21,22]. Infrared autofluorescence was acquired from three locations on the old, mid, and young leaves of plants treated at 4 °C and 25 °C on D9. Autofluorescence was excited with an 830 nm laser and acquired from 860 to 970 nm—these wavelengths are too long for absorption or emission of either chlorophyll a or b [23]. We find that there is an inverse correlation between the infrared autofluorescence and chlorophyll, suggesting that this autofluorescence arises from chlorophyll degradation products. The data were preprocessed and analyzed using Python—the spectra presented are 5th polynomial fits to the acquired spectra.

### 2.4. Determination of Total Nitrogen Content

For the measurement of total N content, we used the organic elemental analyzer vario EL cube (Elementar, Langenselbold, Germany). Briefly, the old, mid, and young leaves were dried at 65 °C and ground into a fine powder, and the analysis was based on combustion, during which the samples generated gases like CO_2_, H_2_O, NO_2_, SO_2_, etc. These gases were then absorbed and separated using chromatography columns and analyzed with thermal conductivity detectors.

### 2.5. Determination of Nitrate Content

The nitrate content in the leaves was measured using the method described previously [21,24].

### 2.6. Determination of Total Protein Content

To determine the total protein contents, the leaves were ground in liquid nitrogen into a fine powder, dissolved in a lysis buffer (50 mM Tris-HCl pH 8.0, 150 mM NaCl, 1% NP-40, protease inhibitor cocktail), and incubated on ice for 30 min. The tubes were then centrifuged at 14,000 rpm, 4 °C for 10 min; the supernatant was mixed with Bradford solution (Bio-Rad, Hercules, CA, USA); and the absorbance was recorded at 595 nm. The standard curve constructed using known concentrations of bovine serum albumin was used to calculate the total amount of protein in samples.

### 2.7. RNA Isolation and Quantitative Real-Time PCR Analysis

The total RNA of Pak Choi leaves was extracted using the FavorPrep^TM^ Plant Total RNA Purification Kit (Favorgen, Ping Tung, Taiwan) according to the manufacturer’s instructions. RNA samples were prepared DNA-free, and the quality was evaluated using an Agilent 2100 Bioanalyzer (Agilent Technologies, Santa Clara, CA, USA). RNA with an RIN value of >7.0 was then reverse transcribed to cDNA, using the iScript™ cDNA Synthesis Supermix (Bio-Rad, Hercules, CA, USA), following the manufacturer’s instructions. PCR primers were designed using Primer3, and qRT-PCR was performed using a Bio-Rad CFX96 Connect real-time PCR instrument and SsoAdvanced Universal SYBR Green Supermix (Bio-Rad, Hercules, CA, USA). *Actin* was used as the control. Primers are listed in Supplementary Table S1. Old leaves from plants stored at 25 °C for 9 days (D9) were omitted from the qRT-PCR assay, as the RIN values were always much lesser than 7.0.

### 2.8. Statistical Analysis

All results were expressed as the mean ± standard deviation (SD). Six biological replicates and three technical replicates were used for all experiments. The statistical analysis was performed using a two-tailed *t*-test in Microsoft Excel.

## 3. Results and Discussion

### 3.1. Effect of Post-Harvest Storage Temperature on Leaf Senescence

Leaf senescence is a critical developmental sequence in plants, as it is aligned with their resource management strategies. Plants have evolved to adopt programmed cell death and senescence to handle unpredictable nutrient shortages. Leaf senescence particularly focuses on the elimination of inefficient and aging photosynthetic organs, which constitute the plant’s nutrient storage [25,26,27]. The orchestration of this process responds to the plant’s source/sink needs and integrates growth hormones, nutrient recognition, and stress response networks.

The storage temperature plays a huge role in maintaining the quality of the leafy vegetables, which are highly perishable. The optimal temperature to achieve the best shelf life for many perishable produces is approximately 0 °C, and every 10 °C increase decreases the shelf life by 2-to-3-fold. In strawberries, low temperature storage delayed leaf aging and prolonged shelf life [28]. The effect of two post-harvest storage temperatures, 4 °C and 25 °C, on leaf senescence over time is illustrated with data in Pak Choi plants (Figure 1B,C). At 25 °C, the senescence rate was much higher than at 4 °C. The old leaves showed yellowing on D3 and by D9, and all but the youngest leaves were completely yellow. The old leaves were partially degraded on D9 at 25 °C. On the other hand, the plants stored at 4 °C exhibited delayed senescence and remained green, except for the few old leaves that showed signs of yellowing on D9 (Figure 1B,C). The chlorophyll analysis and the infrared autofluorescence measurements correlated with the morphological phenotype in that the total chlorophyll content decreased rapidly with time in plants stored at 25 °C (Figure 1C and Supplementary Figure S1). Contrastingly, the chlorophyll content was significantly higher even on D9 in plants stored at 4 °C compared to those stored at 25 °C (Figure 1C). In summary, the cold temperature delayed senescence and preserved the visual and textural quality of the Pak Choi plants.

### 3.2. Effect of Post-Harvest Storage Temperature on Total Nitrogen, Nitrate, and Protein Content in Leaves

**Figure 2:**
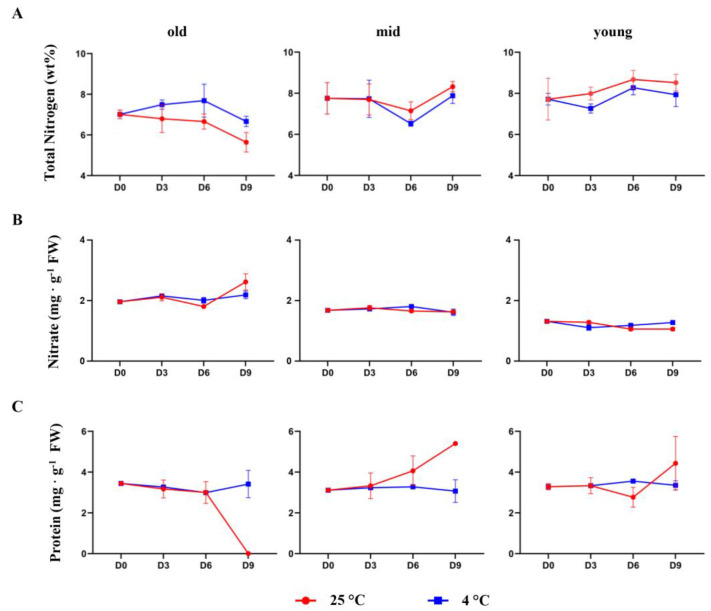
Effect of the post-harvest storage temperature on total nitrogen, nitrate, and protein contents in Pak Choi leaves. Total nitrogen content (**A**), nitrate content (**B**), and protein content (**C**) in old (leaves 1–4), mid (leaves 5–8), and young (leaves 8–12) leaves were analyzed on the harvesting day (D0) and on day 3 (D3), day 6 (D6), and day 9 (D9) after storage at 4 °C and 25 °C under dark conditions. Data are mean ± SD, n = 6. The statistical significance of the 4 °C treatment compared to the 25 °C treatment was determined by a two-tailed *t*-test, and the *p*-values are given in Supplementary Table S3.

N stands out as a pivotal element that is remobilized in massive quantities during natural senescence [5]. To understand more about N assimilation and remobilization during post-harvest leaf senescence, we checked the levels of total nitrogen, nitrate, and protein content in old, mid, and young leaves of Pak Choi plants on D0 and on D3, D6, and D9 after storage at 4 °C and 25 °C. In the old leaves of plants stored at 25 °C, the total N content decreased persistently with time (Figure 2A). Inversely, in the young leaves of the same plants, the total N content increased until D6 and remained high on D9. In the middle leaves, the levels of total N remained nearly constant throughout the study period. Consistent with the lack of leaf senescence and obviating the need for N remobilization, the reverse trend could be observed in plants stored at 4 °C - the total N content increased in the old leaves with time. On D9 of 4 °C storage, the total N content in the old leaves showed signs of decreasing, which might be indicative of the initiation of leaf senescence (Figure 2A).

The N remobilization from senescing leaves depends, to an uncertain extent, on inorganic N molecules such as nitrate, ammonium, and urea [29]. The role of these inorganic forms is contingent on the feeding patterns of the plants and their storage of inorganic N in vegetative parts prior to senescing. To understand if the changes in the total N content were brought about by nitrate remobilization, we measured the total nitrate content at different leaf positions and time points. The results revealed that the post-harvest storage temperature did not affect the nitrate content in the leaves, suggesting the absence of nitrate ion remobilization (Figure 2B).

During leaf senescence, chloroplasts and proteins degrade, releasing nutrients like sugars, lipids, and amino acids, which are then remobilized. Previous studies have identified specific amino acids, notably glutamine, serine, asparagine, glutamate, and aspartate, as predominant in phloem sap; however, variations may occur depending on the plant species [30,31,32,33]. To investigate if amino acid transport facilitated the changes in the total N content in Pak Choi leaves, we measured the total protein content in the different leaf groups on different days. Notably, in plants stored at 25 °C, the old leaves exhibited a marked decrease in protein content, possibly linked to chloroplast protein degradation (Figure 2C). A decrease in protein content is not unique to senescing Pak Choi leaves; in fact, a similar study in strawberries reported that the storage of post-harvest fruits at a high temperature decreased the protein content, thus impairing their photosynthetic ability [28]. Interestingly, in plants stored at 25 °C, the total protein content in the middle leaves began to increase steadily from D3, while, in the young leaves, it showed an increase on D9. It is important to note that the total protein content remained unchanged across all leaves on all days in plants stored at 4 °C (Figure 2C), reinforcing the notion of negligible chloroplast breakdown and, therefore, delayed senescence.

### 3.3. Effect of Post-Harvest Storage Temperature on Transcript Levels of Genes Involved in Nitrogen Assimilation

**Figure 3:**
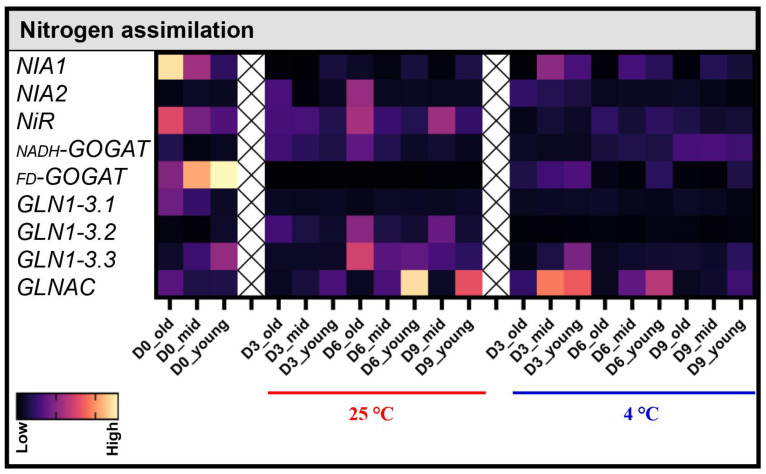
Effect of the post-harvest storage temperature on transcript levels of genes involved in nitrogen assimilation in Pak Choi leaves. Transcript levels of genes involved in N assimilation in old (leaves 1–4), mid (leaves 5–8), and young (leaves 8–12) leaves were analyzed on the harvesting day (D0) and on day 3 (D3), day 6 (D6), and day 9 (D9) after storage at 4 °C and 25 °C under dark conditions. The transcript levels shown are relative to the mRNA levels of *Actin*. Data are mean ± SD, n = 6. The statistical significance of the 4 °C treatment compared to the 25 °C treatment was determined by a two-tailed *t*-test, and the *p*-values are given in Supplementary Table S4.

Once nitrate transporters import nitrate ions from roots, nitrate reductases convert these ions into nitrite ions, which are then converted into ammonium ions by nitrite reductases. Since these genes play a critical role in N assimilation in plants, we examined their transcript levels via qRT-PCR. It is important to note that the old leaves from plants that were stored at 25 °C for 9 days (D9 old) were not included in the qRT-PCR study, as the quality of the RNA isolated from those samples was poor. Differentially expressed genes between 4 °C and 25 °C are shown in Figure 3. Upon analyzing the expression of nitrate reductases and nitrite reductases, we observed that the nitrate reductase *NIA1* was highly expressed in the old and mid leaves on D0. However, its transcripts were completely suppressed in all leaves stored at 25 °C from D3 (Figure 3). In contrast, a delay in the suppression of *NIA1* transcripts was detected in plants stored at 4 °C. Interestingly, *NIA2* was not expressed on D0, but its expression began to increase gradually in old leaves stored at 25 °C on both D3 and D6. It is important to note that, in Arabidopsis, of the two *NIA* genes, *NIA1* was highly upregulated and *NIA2* remained unchanged during natural leaf senescence [34], whereas, in post-harvest senescent Pak Choi leaves, the mRNA levels of *NIA1* were found to be decreased, and mRNA levels of *NIA2* were increased.

During natural senescence, nitrite reductase *NiR* was found to be downregulated in Arabidopsis [34]. In Pak Choi, *NiR* transcripts were detected in the old leaves on D0 but were found reduced upon storage at 4 °C, similar to Arabidopsis natural senescence. However, at 25 °C storage, the expression levels of *NiR* increased from D6 in both old and mid leaves (Figure 3).

Glutamine and asparagine, both containing two N atoms per molecule, are the favored export forms in plants. In young leaves, glutamine is primarily synthesized within the chloroplast by glutamine synthetase (GS2) and ferredoxin-dependent glutamate synthase (FD-GOGAT). However, as chloroplasts degrade during leaf senescence, the localization of glutamine synthetase shifts, with GS activity transitioning to the cytosol, where GS1 compensates for the reduced GS2 activity [29,35]. When we investigated the mRNA levels of glutamate synthases and glutamine synthetases, we observed contrasting patterns. Specifically, *NADH-GOGAT*, which usually participates in ammonium assimilation in roots [36], was not expressed on D0, and it showed slight induction in old leaves during storage, whereas *FD-GOGAT* was highly expressed in the mid and young leaves on D0, but its transcript levels decreased during storage at both 4 °C and 25 °C (Figure 3). The mRNA levels of the chloroplastic *GS2/GLNAC* were interesting, as chloroplastic *GS2/GLNAC* was already absent in the old leaves stored at 25 °C on D3, indicating the onset of senescence and degradation of chloroplast proteins. In contrast, these transcripts were undetected only on D6 in the old leaves stored at 4 °C (Figure 3). Additionally, *GLNAC* was induced in the young leaves during post-harvest storage at both 4 °C and 25 °C. These results showed that the expression patterns of *NADH-GOGAT*, *FD-GOGAT*, and *GNLAC* during post-harvest leaf senescence in Pak Choi were analogous to their expression patterns during natural senescence in Arabidopsis [34].

Although Arabidopsis cytosolic glutamine synthetases were upregulated during natural senescence [34], transcripts of Pak Choi *GS1/GLN1-3.1* were found in low levels in the old leaves on D0 and were completely suppressed upon post-harvest storage at both 4 °C and 25 °C (Figure 3). While transcripts of *GLN1-3.2* were not detected on D0, transcripts of *GLN1-3.3* were detected in the young leaves of D0 samples. Interestingly, both *GLN1-3.2* and *GLN1-3.3* showed increased mRNA levels in the old leaves of plants stored at 25 °C but not at 4 °C. This suggests the potential involvement of these genes in the remobilization of glutamine and asparagine during post-harvest leaf senescence.

### 3.4. Effect of Post-Harvest Storage Temperature on Expression of Genes Involved in Nitrogen Remobilization

**Figure 4:**
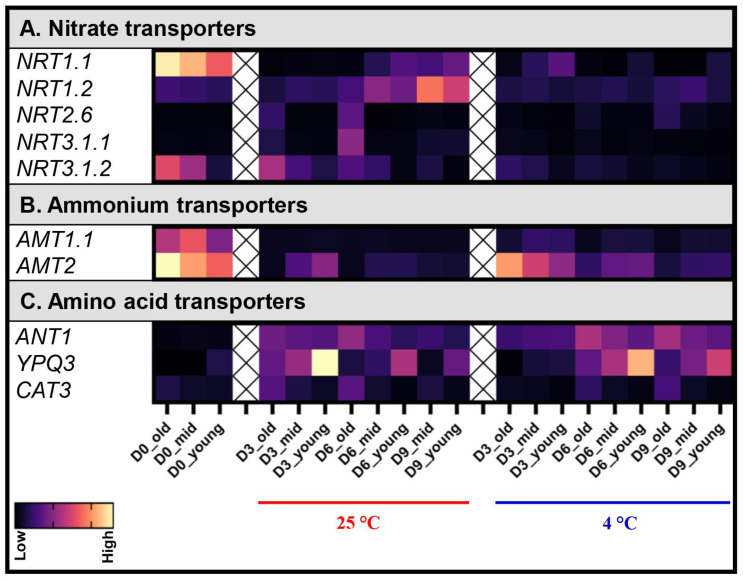
Effect of the post-harvest storage temperature on transcript levels of genes involved in nitrogen remobilization in Pak Choi leaves. Transcript levels of genes involved in N remobilization in old (leaves 1–4), mid (leaves 5–8), and young (leaves 8–12) leaves were analyzed on the harvesting day (D0) and on day 3 (D3), day 6 (D6), and day 9 (D9) after storage at 4 °C and 25 °C under dark conditions. The expression levels shown are relative to the expression of *Actin*. Data are mean ± SD, n = 6. The statistical significance of the 4 °C treatment compared to the 25 °C treatment was determined by a two-tailed *t*-test and the *p*-values are given in Supplementary Table S5.

The nitrate transporters *NRT1.1*, *NRT1.5*, *NRT1.6*, and *NRT2.5* were upregulated, and *NRT1.11*, *NRT1.12*, and *NPF5.10* were downregulated, during natural senescence in Arabidopsis [34]. Among these genes, only transcripts of *NRT1.1* were found in Pak Choi leaves during post-harvest senescence. High levels of *NRT1.1* mRNA were detected in the D0 leaf samples. However, its transcript levels dropped drastically from D3 to D9 samples at both 4 °C and 25 °C (Figure 4A). This suggests that NRT1.1 plays a role in importing nitrate ions from the roots to the shoots under pre-harvest conditions but becomes inactivated in leaf samples post-harvest. The advanced understanding of nitrate signaling pathways has revealed that master transcription factors active downstream of NRT1.1, involved in the regulation of crucial nitrate-responsive genes like *NIA*, *NiR*, and *NRT2.1* [37]. In our study, the mRNA levels of *NRT1.1* correlates with that of the nitrate reductase *NIA1* and the nitrite reductase *NiR* only on D0. This implies that NRT1.1 might not regulate nitrate-responsive genes during post-harvest leaf senescence (Figure 3 and Figure 4A).

On the other hand, *NRT1.2* had a low expression in D0 samples and in all samples stored at 4 °C. But in samples stored at 25 °C, its expression was significantly induced by D9, as shown in Figure 4A. Once again, the mRNA levels of *NRT1.2* under post-harvest conditions were different from natural senescence, where it was unaffected [34]. Furthermore, *NRT2.6* and *NRT3.1* were differentially expressed in senescing leaves (Figure 4A). While NRT2.6 has been shown to mediate nitrate transport in Xenopus oocytes [38], its specific functions in planta remain undefined. An association between AtNRT2.6 activity and the production of reactive oxygen species has been observed in response to the redox-active herbicide methyl viologen, hinting at potential stress-related roles [39]. In plants stored at 25 °C, mRNAs of *NRT2.6* and *NRT3.1.1* were not detected on D0, but in post-harvest plants, they were exclusively induced in the old leaves on D3 and D6 (Figure 4A). However, *NRT3.1.2* showed a high expression in D0 samples, and their transcript levels were drastically reduced upon storage at both 4 °C and 25 °C (Figure 4A). Unlike natural senescence conditions, a key common observation under post-harvest conditions is that most nitrate transporters are deactivated, which might be attributed to the absence of the remobilization of the nitrate ion.

Along with nitrate ions, ammonium ions are also thought to be remobilized during natural leaf senescence with the aid of ammonium transporters. *AMT1.5* has been shown to be expressed in senescing leaves [3]. When we examined the ammonium transporters in post-harvest leaves, transcripts of *AMT1.5* were not detected in Pak Choi leaves. Instead, mRNAs of *AMT1.1* and *AMT2* were found highly in all leaves on D0. Even though their levels remained high in samples stored at 4 °C on D3, transcript levels of *AMT1.1* and *AMT2* decreased drastically in plants stored at 25 °C. By D6 and D9, the mRNAs of *AMT1.1* and *AMT2* were extremely less in all samples (Figure 4B), suggesting that the ammonium ions might not be remobilized during post-harvest leaf senescence in Pak Choi. The reduction in the mRNA levels of *AMT1.1* and *AMT2* in Pak Choi during post-harvest leaf senescence is different from that of the Arabidopsis study, where they remained unchanged during natural senescence [34].

Despite inorganic nitrogen’s role in source/sink remobilization, the extent of its contribution remains ambiguous. Given the relative scarcity of nitrate and ammonium compared to protein N sources, the major remobilization might involve proteolysis, amino acid interconversions, and translocation to the phloem sap [32,40]. An interesting observation concerns the upregulation of several AAP amino acid permeases during senescence, notably AAP2 and AAP8, both of which have been associated with source/sink N remobilization [10,41]. Arabidopsis mutants lacking the *aap2* gene manifested altered developmental traits, such as delayed leaf senescence and increased seed yield, although with modified N and fatty acid content, indicating the control of sink/source relations by AtAAP2 [10]. AAP8 is particularly important in N transfer to seeds, with alterations in leaf N pools in *aap8* mutants resulting in reduced silique and seed numbers [9]. Surprisingly, the Arabidopsis orthologs of *AAP2* and *AAP8* were not found in Pak Choi leaves during post-harvest storage. This raises questions whether these AAP amino acid permeases function exclusively during natural leaf senescence. In contrast, transcript levels of three amino acid transporters, *ANT1*, *CAT3*, and *YPQ3*, increased in Pak Choi leaves during post-harvest senescence (Figure 4C). The AAP amino acid permease *ANT1* and cationic amino acid transporter *CAT3* were induced in old leaves starting from D3 at 25 °C and from D6 upon storage at 4 °C. *ANT1* and *CAT3* have also been reported to be expressed during natural leaf senescence in Arabidopsis [34]. Interestingly, the probable vacuolar amino acid transporter gene *YPQ3*, which remains functionally uncharacterized in planta, emerged as the most induced gene in young leaves on D3 and D6 when stored at 4 °C and 25 °C, respectively (Figure 4C). A more comprehensive functional characterization of YPQ3 might unveil its role in the remobilization of amino acids during post-harvest leaf senescence.

### 3.5. Effect of Post-Harvest Storage Temperature on NLA and SIZ1—Regulators of Nitrogen-Dependent Leaf Senescence

Previous studies reported that an E3 ubiquitin ligase NITROGEN LIMITATION ADAPTATION (NLA) and an E3 small ubiquitin-related modifier (SUMO) ligase SIZ1 regulate the leaf senescence process under N deficiency and N assimilation, respectively [42,43]. In Arabidopsis, SIZ1 SUMOylated and dramatically increased the activities of NIA1 and NIA2 [42], whereas NLA regulated the stability of ORE1 through polyubiquitination [43].

**Figure 5:**
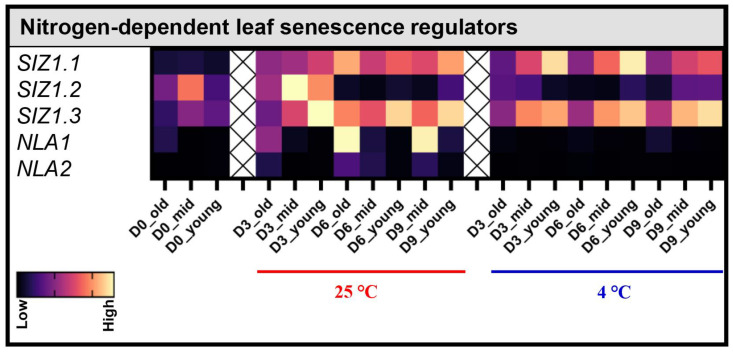
Effect of the post-harvest storage temperature on transcript levels of nitrogen-dependent leaf senescence regulators SIZ1 and NLA. Transcript levels of genes involved in regulation of leaf senescence in old (leaves 1–4), mid (leaves 5–8), and young (leaves 8–12) leaves were analyzed on the harvesting day (D0) and on day 3 (D3), day 6 (D6), and day 9 (D9) after storage at 4 °C and 25 °C under dark conditions. The expression levels shown are relative to the expression of *Actin*. Data are mean ± SD, n = 6. The statistical significance of the 4 °C treatment compared to the 25 °C treatment was determined by a two-tailed *t*-test, and the *p*-values are given in Supplementary Table S6.

In Pak Choi, among differentially expressed *SIZ1* genes, *SIZ1.1* was not detected on D3, whereas *SIZ1.2* and *SIZ1.3* showed low mRNA levels on the same day (Figure 5). Interestingly, during post-harvest storage, the transcript levels of *SIZ1.1* and *SIZ1.3* significantly increased in both mid and young leaves at both 4 °C and 25 °C. In contrast, the mRNA levels of *SIZ1.2* increased only at 25 °C on D3, and they decreased on the subsequent days and when stored at 4 °C. The qRT-PCR analysis also revealed that the mRNA levels of *NLA1* genes were specifically increased in the old leaves stored at 25 °C (Figure 5). This suggests that NLA1 might be involved in a N-dependent regulation during post-harvest leaf senescence.

## 4. Conclusions

The post-harvest storage temperature exerts a significant influence on leaf senescence, nitrogen content, protein degradation, and gene expression in Pak Choi plants. At a higher storage temperature (25 °C), the rate of senescence is accelerated, marked by the yellowing of leaves and a rapid decrease in chlorophyll content. At a lower temperature (4 °C), however, senescence is delayed, preserving the visual and textural quality of the leaves.

Furthermore, our study illustrated the differential response of total nitrogen, nitrate, and protein content based on storage temperature. N remobilization appeared to be altered by storage conditions, impacting both young and old leaves differently. Key transporters and enzymes involved in N assimilation and remobilization were also impacted by temperature. Intriguingly, certain transporters (e.g., AAP2 and AAP8) found in other species, like Arabidopsis, were absent in Pak Choi under post-harvest conditions, suggesting unique senescence pathways or potential redundancies in Pak Choi. The expression patterns of the nitrate transporters and associated genes, especially under 4 °C storage conditions, highlighted a deactivated nitrate remobilization mechanism, which contrasts with natural senescence conditions.

Given the intricate orchestration of processes, we can expect that optimizing post-harvest storage conditions will significantly extend the shelf life of leafy vegetables, such as Pak Choi. Furthermore, understanding the molecular mechanisms underlying post-harvest senescence can inform agricultural practices and post-harvest management to minimize nutrient loss and maintain product quality. From our observations, it is also anticipated that different leafy vegetables might exhibit unique patterns of N remobilization and protein degradation, which can open avenues for species-specific post-harvest interventions.

## Data Availability

All data supporting the findings of this study are available within the paper and its Supplementary Information.

## Supplementary Materials

The following supporting information can be downloaded at https://www.mdpi.com/article/10.3390/biom13101540/s1, Figure S1: Effect of post-harvest storage temperature on fluorescence intensity in Pak Choi leaves; Table S1: List of primers used in the qRT-PCR; Table S2: The *p*-value data for Figure 1C; Table S3: The *p*-value data for Figure 2; Table S4: The *p*-value data for Figure 3; Table S5: The *p*-value data for Figure 4; Table S6: The *p*-value data for Figure 5.
